# Dental follicle mesenchymal stem cell administration ameliorates muscle weakness in MuSK-immunized mice

**DOI:** 10.1186/s12974-015-0451-0

**Published:** 2015-12-09

**Authors:** Canan Ulusoy, Noushin Zibandeh, Selin Yıldırım, Nikolaos Trakas, Paraskevi Zisimopoulou, Melike Küçükerden, Hatice Tașlı, Socrates Tzartos, Kamil Göker, Erdem Tüzün, Tunç Akkoç

**Affiliations:** Department of Neuroscience, Institute for Experimental Medical Research (DETAE), Istanbul University, Istanbul, Turkey; Division of Pediatric Allergy and Immunology, Marmara University Faculty of Medicine, Istanbul, Turkey; Department of Neurobiology, Hellenic Pasteur Institute, Athens, Greece; Department of Oral and Maxillofacial Surgery, Marmara University Faculty of Dentistry, Istanbul, Turkey; Department of Neurology, Istanbul Faculty of Medicine, Istanbul University, Istanbul, Capa Turkey

**Keywords:** Myasthenia gravis, Muscle-specific kinase, Mesenchymal stem cells, Dendritic cells, Autoimmunity

## Abstract

**Background:**

Myasthenia gravis (MG) is an antibody-mediated autoimmune disease of the neuromuscular junction (NMJ), mostly associated with acetylcholine receptor (AChR) antibodies. Around 5–10 % of MG patients show antibodies to muscle-specific tyrosine kinase (MuSK). Mesenchymal stem cell (MSC) administration has been shown to ameliorate muscle weakness in the experimental autoimmune myasthenia gravis (EAMG) model induced by AChR immunization.

**Methods:**

To investigate the efficacy of stem cell treatment in MuSK-related EAMG, clinical and immunological features of MuSK-immunized mice with or without dental follicle MSC (DFMSC) treatment were compared.

**Results:**

MuSK-immunized mice intravenously treated with DFMSC after second and third immunizations showed significantly lower EAMG incidence and severity and reduced serum anti-MuSK antibody, NMJ IgG, and C3 deposit levels and CD11b+ lymph node cell ratios. Moreover, lymph node cells of DFMSC-administered mice showed reduced proliferation and IL-6 and IL-12 production responses to MuSK stimulation. By contrast, proportions of B and T cell populations and production of a wide variety of cytokines were not affected from DFMSC treatment.

**Conclusions:**

Our results suggest that DFMSC treatment shows its beneficial effects mostly through suppression of innate immune system, whereas other immune functions appear to be preserved. Stem cell treatment might thus constitute a specific and effective treatment method in MuSK-associated MG.

**Electronic supplementary material:**

The online version of this article (doi:10.1186/s12974-015-0451-0) contains supplementary material, which is available to authorized users.

## Background

Myasthenia gravis (MG) is a T cell-dependent and antibody-mediated autoimmune disease characterized with fluctuating muscle weakness due to neuromuscular junction (NMJ) dysfunction mostly induced by acetylcholine receptor (AChR) antibodies [1]. MG is currently treated with global immunosuppressants with substantial side effects including steroids, azathioprine, and other cytotoxic drugs. Around 5–10 % of MG patients display muscle-specific tyrosine kinase (MuSK) antibodies. MuSK antibody-positive MG patients often present with a severe clinical course and higher dosages of immunosuppressants are required for their management [2], prompting the innovation of novel therapeutic reagents with specific mechanisms of action and fewer side effects.

Mesenchymal stem cells (MSCs) are multipotent adult stem cells. They have been isolated from various sources, such as cord blood, Wharton’s jelly, the placenta, bone marrow, teeth, and adipose tissue [3–11].

A promising source of MSCs is dental tissue, which is easily accessible and can be isolated from many sources of the orofacial region, such as stem cells isolated from human exfoliated deciduous teeth (SHEDs), dental pulp stem cells (DPSCs), dental follicle stem cells (DFSCs), and periodontal ligament stem cells (PDLSCs) [11–14]. MSCs show expression of embryonic stem cell markers Oct4, NANOG, SOX2, alkaline phosphatase, and SSEA-4 in adult MSC populations derived from the bone marrow, adipose tissue, dermis, and heart [9–14].

As a promising therapeutic tool to suppress inflammation and immunomodulation, MSCs have been widely used in preclinical treatment studies of several autoimmune disorders [15–21]. Recently, MSCs from bone marrow have been successfully employed in AChR-induced experimental autoimmune myasthenia gravis (EAMG) model resulting in amelioration of muscle weakness and reduction of AChR-reactive lymphocytes [21]. However, in this previous study, immunopathogenic aspects of EAMG have not been comprehensively investigated.

In this study, the efficacy of stem cell treatment has been tested in MuSK-associated EAMG for the first time and immunopathogenic features of MSC-treated mice have been analyzed. Our results suggest that MSC administration might constitute a specific and effective treatment method in MuSK-associated MG.

## Methods

### Mice and MuSK

Seven- to 8-week-old wild-type C57BL/6 (B6) mice were purchased from Jackson Laboratories (Bar Harbor, ME, USA). All animals were housed in the viral antibody-free barrier facility at the Istanbul University and maintained according to the Institutional Animal Care and Use Committee Guidelines. The extracellular domain of human MuSK (amino acids 1–463, MUSK_HUMAN O15146-3) was cloned into the pPICZαA vector (Invitrogen, San Diego, CA, USA) and was expressed in Pichia pastoris host strain X33 as soluble protein in the yeast culture supernatant as described previously [22, 23]. The expressed protein was purified by metal affinity chromatography using Ni-NTA agarose resin (Qiagen, Valencia, CA, USA), according to the manufacturer’s protocol. The purity of the protein was documented by gel electrophoresis and western blotting with a commercial anti-human MuSK antibody (Abcam, Cambridge, UK).

### Isolation of dental follicle MSC (DFMSC)

Dental follicles (DF) were collected from the Marmara University Faculty of Dentistry Oral and Maxillofacial Surgery. The legitimate delegate of all patients provided informed consent according to the guidelines of the Ethics Committee of the Marmara University Medical Faculty in Istanbul, Turkey (09.2014.0015/70737436-050.06.04). These follicles were transported in Dulbecco’s phosphate-buffered saline (DPBS, Gibco, Grand Island, NY 14072, USA) containing 1 % penicillin/streptomycin (Gibco, USA). All laboratory work was performed in a laboratory in the Department of Pediatric Allergy-Immunology, Marmara University Research Hospital.

Follicles were isolated under sterile conditions. They were enzymatically treated with 3 mg/ml collagenase type I (Gibco, USA) for 45 min at 37 °C to completely digest pulp and follicle tissue. Then, 3 ml of Dulbecco’s modified Eagle’s medium (DMEM, Gibco, USA) supplemented with 10 % fetal bovine serum (FBS, Gibco, USA) and 1 % penicillin/streptomycin was added to digest the pulp and follicle tissue followed by centrifugation at 1200 rpm for 5 min. Cell pellets were obtained, and the supernatant was aspirated. DFMSCs were cultivated in T-25 flasks in a 5 % CO_2_ atmosphere under 37 °C in culture medium composed of DMEM, 10 % FBS, and 1 % penicillin/streptomycin. The stem cells were washed with DPBS and provided with fresh culture medium. The culture medium was changed every 3 to 4 days until the cells reached confluence. The cells were detached with 0.25 % trypsin-EDTA (Gibco, USA) when they reached 70–80 % confluence. Adherent cells cultured for 3 passages were characterized and analyzed for specific surface markers. The cellular analyses and differentiation were performed using flow cytometry.

### Flow cytometry analysis of DFMSCs

To analyze the cell surface antigen expressions, the cells from the third passage were used. DFMSCs were incubated with antibodies for human CD73 phycoerythrin (PE), CD90 PE, CD146 fluorescein isothiocyanate (FITC), CD29 allophycocyanin (APC), CD105 PE, CD45 FITC, CD34 PE, CD14 PE, CD25 APC, and CD28 PE (BD Biosciences, San Diego, CA, USA) at room temperature in the dark. Control antibodies were PE-conjugated or FITC-conjugated and APC-conjugated mouse IgG1 and mouse IgG2 (BD Biosciences, San Diego, CA, USA). The flow cytometry results were analyzed using BD FACS Calibur.

### Differentiation of stem cells

To induce osteogenic (MesenCult, Stemcell Technologies, North America), adipogenic, and chondrogenic differentiation, a human MSC functional identification kit (Gibco, Grand Island, USA) was used. For differentiation, the cells were plated in 6-well plates (5 × 10^4^ cell/well), and the differentiation medium was prepared according to the manufacturer’s instructions and changed 3 times per week. After 14 days, the adipocytes and chondrocytes were stained with Oil Red O and Alcian blue, respectively, and after 28 days, the osteocytes were stained with Alizarin red [24].

### Real-time PCR analysis

Total RNA was isolated from 1 × 10^6^ DFMSCs at passage 3 using a high pure RNA isolation kit (Roche, Mannheim, Germany) according to the manufacturer’s instructions. One microgram of total RNA was converted to cDNA using a Transcriptor first-strand cDNA synthesis kit (Roche Mannheim, Germany). Equal amounts of cDNA were used for the real-time amplification of the target genes according to the manufacturer’s recommendations using a LightCycler 480 Real-Time PCR System (Roche Diagnostic, Mannheim, Germany). The gene expression of specific markers for MSCs, including alkaline phosphatase (ALPL), runt-related transcription factor 2 (RUNX2), NANOG, NESTİN, NOTCH, and dentin sialophosphoprotein (DSPP), was quantified relative to the housekeeping gene glyceraldehyde 3-phosphate dehydrogenase (GAPDH). The RT-PCR conditions were as follows: pre-incubation for 10 min at 95 °C for 1 cycle; amplification for 10 s at 95 °C, 60 °C for 30 s, 72 °C for 1 s for 45 cycles; and cooling for 10 s at 40 °C for 1 cycle. The reaction mixture lacking cDNA was used as a negative control in each run. The real-time PCR results were analyzed using LightCycler software (version 2).

### Induction and clinical evaluation of EAMG

A total of 20 B6 mice were anesthetized and immunized with 30 μg of MuSK emulsified in complete Freund’s adjuvant (CFA, Difco, Detroit, MI, USA) s.c. at four sites (two hind footpads and shoulders) on day 0 and were boosted with the same amount of MuSK in CFA s.c. at four sites on the back on days 28 and 56. An additional 20 control mice were immunized with only CFA. The number of cells to be administered per injection was determined during optimization studies. During these studies, clinical results obtained with mouse compact bone MSC and DFMSC treatments were also compared (Additional file 1: Table S1). The DFMSCs were freshly prepared before each injection. They were trypsinized and washed for two times with PBS, and 1 × 10^6^ DFMSCs were administered intravenously in 1 h via an insulin syringe from the tail vein twice (7 days after second and third immunizations) to MuSK + CFA (MuSK-SC, *n* = 10) and only CFA (CFA-SC, *n* = 10) -immunized mice. The remaining mice from MuSK + CFA (*n* = 10) and only CFA (n = 10) groups were used as non-DFMSC treatment controls and were treated with PBS only. Mice were terminated 28 days after the third immunization.

For clinical examination, mice were left for 3 min on a flat platform and were observed for signs of EAMG. Clinical muscle weakness was graded as follows: grade 0, mouse with normal posture, muscle strength, and mobility; grade 1, normal at rest, with muscle weakness characteristically shown by a hunched posture, restricted mobility and difficulty raising the head after exercise that consisted of 30 paw grips on a cage top grid; grade 2, grade 1 symptoms without exercise during the observation period on a flat platform; grade 3, dehydrated and moribund with grade 2 weakness; and grade 4, dead.

Inverted screen test was administered for quantitative evaluation of muscle weakness. Mice were placed in the center of a screen of wire mesh, which was immediately rotated to the inverted position and held steadily 50 cm above a padded surface. The time at which the mouse fell off was noted with an endpoint of 300 s. The recorded time was compared for statistical significance among treatment and control groups.

### ELISA for anti-MuSK Ig isotypes

Mice were bled from the tail vein during termination. Sera were evaluated for anti-MuSK IgG, IgG1, IgG2b, IgG3, and IgM levels. Affinity-purified human MuSK (1 μg/ml) was coated onto 96-well microtiter plates in 0.1 M carbonate bicarbonate buffer overnight at 4 °C. Diluted serum samples of 100 μl (1:1000) were added and incubated at 37 °C for 90 min. Horseradish peroxidase (HRP)-conjugated anti-mouse IgG, IgG1, IgG2b, IgG3, and IgM (Abcam) (1:10,000) were added and then incubated at 37 °C for 90 min. Subsequently, the peroxidase indicator substrate 2,2′-azinobis-(3-ethylbenzothiazoline 6-sulfonate) substrate (ABTS) solution in 0.1 M citric buffer (pH 4.35) was added in the presence of H_2_O_2_, and the mixture was allowed to develop color at room temperature in the dark. Plates were read at a wavelength of 405 nm.

### Immunofluorescence for NMJ IgG and C3 deposits

Sections (10 μm thick) were obtained from forelimb muscle samples of mice, frozen in liquid nitrogen, and stored at −80 °C. Slides were fixed in cold acetone and blocked in 10 % normal goat serum in PBS. After washing with PBS, the sections were incubated with tetramethylrhodamine-conjugated bungarotoxin (BTx) (Molecular Probes, Eugene, OR) (1/500 dilution) for 1 h at room temperature to label the NMJ. Sections were then incubated for 1 h at room temperature with FITC-conjugated antibodies to mouse IgG or complement factor C3 (Abcam) (diluted 1/1000) to colocalize IgG and C3 deposits in NMJ. The sections were washed and viewed in a fluorescence microscope (Olympus IX-70). The number of IgG- and C3-positive BTx binding sites was counted in five muscle sections from each mouse. The percentages of NMJs with deposits in each muscle section were calculated by totaling the numbers of deposits divided by the numbers of BTx-labeled sites, times 100.

### Flow cytometry for lymph node cell subpopulations

In immunization-based EAMG models, antigens are injected subcutaneously to body parts that are in close proximity with the lymph nodes. As a result, immunopathogenic processes leading to antibody formation and muscle weakness predominantly occur in the local draining lymph nodes. Therefore, in our study, all immunopathological studies were conducted using lymph node cells. For this purpose, inguinal, popliteal, and axillary lymph node cells were collected at the termination of the experiment. Single-cell suspensions of lymph node cells were incubated for 30 min with one of the following anti-mouse antibodies: PE-conjugated anti-CD4, anti-CD19, anti-CD11b, and anti-CD3 and FITC-conjugated anti-CD8 (all from BD PharMingen). PE- or FITC-conjugated isotypes were used for controls. Cells were washed twice and then were fixed with 2 % paraformaldehyde and analyzed by flow cytometry (BD Biosciences).

### Lymphocyte proliferation assay

The percentage of proliferating cells in response to MuSK stimulation was measured by carboxyfluorescein succinimidyl ester (CFSE) labeling. Lymph node cells (6 × 10^5^ cells/well) were seeded in triplicate into 48-well plates in 0.5 ml of culture medium [RPMI 1640 supplemented with 10 % fetal calf serum, penicillin G (100 U/ml) streptomycin (100 μg/ml), L-glutamine (2 mM), 2-mercaptoethanol (3 × 10^−5^ M) and HEPES buffer (25 mM)] in the absence or presence (5 or 15 μg) of MuSK. After being cultured for 4 days at 37 °C in humidified 5 % CO_2_-enriched air, cells were labeled with CFSE-FITC (5 μmol/L; Molecular Probes Europe, BV, Leiden, The Netherlands) and analyzed by flow cytometry (BD Biosciences).

### Cytokine measurements in culture supernatants

Lymph node cells (2 × 10^5^ cells/well) were seeded in triplicate into 96-well, round-bottomed microtiter plates in 0.2 ml of culture medium in the absence or presence (5 or 15 μg) of MuSK. The cells were cultured for 48 h at 37 °C in humidified 5 % CO_2_-enriched air. Supernatants were collected and stored at −80 °C until analyzed. The supernatant levels of IL-4, IL-10, IL-13, IFN-γ, IL-12, IL-21, IL-17, IL-6, TGF-β, and TNF-α were measured by ELISA kits (Invitrogen, Carlsbad, CA, USA), according to the manufacturer’s instructions.

### Statistical analysis

Clinical EAMG incidences were compared using the Fisher’s exact test. Clinical grades were compared by Kruskal–Wallis test and Dunn’s post hoc test. All other parameters were compared using ANOVA (and Tukey’s post hoc test). *p* values less than 0.05 were considered statistically significant.

## Results

### Isolation, characterization, and differentiation of DFMSCs

**Fig. 1:**
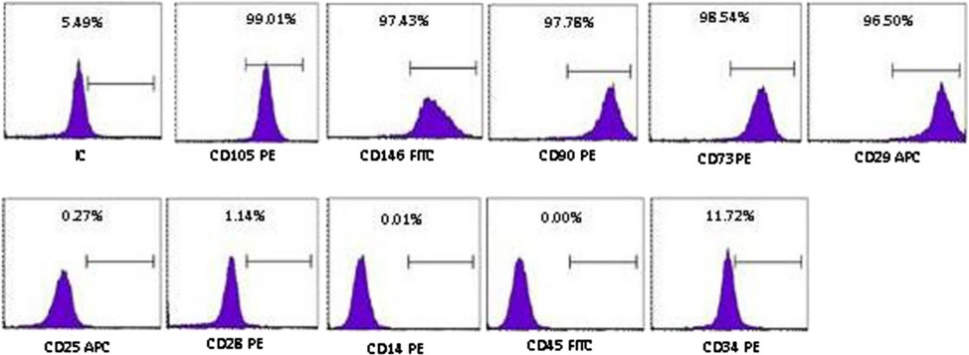
Representative flow cytometry analysis of cell surface markers in dental follicle mesenchymal stem cells (DFMSCs). Representative flow cytometry analysis of cell-surface markers on DFMSCs in P3

DFMSCs attached sparsely to the culture flasks and exhibited a fibroblast-like and spindle-shaped morphology during the early days of incubation. The DFMSCs began to proliferate in approximately 2 days and gradually formed small colonies. The DFMSCs reached 70 % confluency in the primary culture 5–6 days after being plated in their first passages (P1). Most of the DFMSCs exhibited fibroblast-like morphology in the later passages. Then, immunophenotyping and differentiation of the third cell passage were observed. The DFMSCs were analyzed via flow cytometry. These cells exhibited positive staining for CD29, CD73, CD90, CD105, and CD146 but were negative for CD14, CD25, CD28, CD34, and CD45 (Fig. 1).

**Fig. 2:**
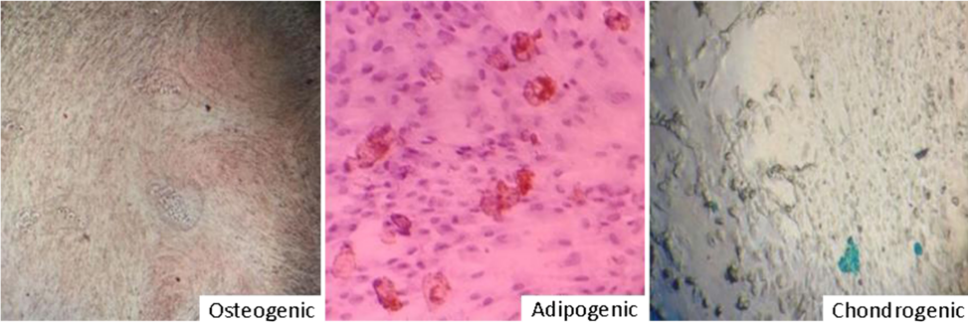
Alizarin red staining of osteogenic-induced dental follicle mesenchymal stem cells (DFMSCs) (*left*), oil red staining of adipogenic-induced DFMSCs (*middle*), and Alcian blue staining of chondrogenic-induced and DFMSCs (*right*) (magnification for all, ×100)

**Fig. 3:**
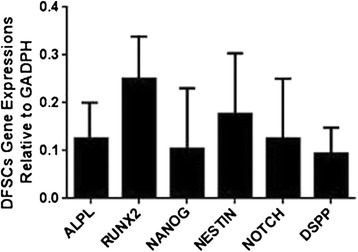
Gene expression of specific markers for dental follicle mesenchymal stem cells (DFMSCs), including alkaline phosphatase (ALPL), runt-related transcription factor 2 (RUNX2), NANOG, NESTİN, NOTCH, and dentin sialophosphoprotein (DSPP) in reference to housekeeping gene glyceraldehyde 3-phosphate dehydrogenase (GAPDH), was performed. *Vertical bars* indicate standard errors

The DFMSCs differentiated into osteocytes, adipocytes, and chondrocytes. First, the osteogenic differentiation of stem cells were evaluated for osteoblast mineralization in the matrix with the stimuli of human osteoblast medium and Alizarin red staining was used for the calcium deposition. The DFMSCs were stained with Alizarin red, and the cells formed calcified bone nodule structures (Fig. 2, left panel). Next, the in vitro adipogenic differentiation capability was assessed by culturing the cells in adipogenic induction medium and staining with Oil Red O. Intracellular lipid droplets were observed in these cells (Fig. 3, middle panel). Finally, the chondrogenic differentiation was evaluated by using Alcian blue. Chondrogenic differentiation medium was used at the end of the culture period. Alcian blue was used to observe proteoglycans in the matrix of cartilage. We observed proteoglycans in blue color in the matrix (Fig. 2, right panel).

We also analyzed the gene expression of specific markers in DFSCs by RT-PCR. The DFSCs expressed ALPL, RUNX2, NANOG, NESTİN, NOTCH, and DSPP genes (Fig. 3).

### DFMSC administration ameliorates clinical symptoms of MuSK-related EAMG

**Fig. 4:**
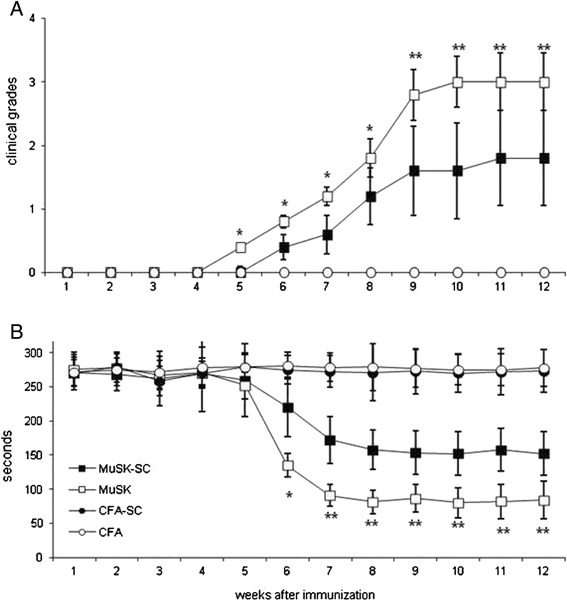
Average clinical grades (**a**) and inverted screen hang times (**b**) of MuSK- and CFA-immunized mice with or without dental follicle mesenchymal stem cell (DFMSC) treatment. MuSK-immunized mice treated with DFMSC (MuSK-SC) showed significantly reduced clinical grades and increased screen hang time than non-treated mice. *Asterisks* only denote significant differences between MuSK and MuSK-SC groups as calculated by post hoc analysis. *p < 0.05; **p < 0.01 by Kruskal–Wallis and Dunn’s post hoc test or ANOVA and Tukey’s post hoc test, as required. *Vertical bars* indicate standard errors. Note that in Fig. 4a, the values of CFA and CFA-SC groups overlap since the average clinical grade for both groups is 0 (no disease) at all time points

At termination, 8 of 10 MuSK-immunized mice and only 4 of 10 of the MuSK-SC group had developed myasthenic muscle weakness (grade ≥ 1) (*p* = 0.034). Moreover, starting from week 5 after first immunization, mice from the MuSK-SC group displayed significantly lower average clinical grades than MuSK-immunized mice with no DFMSC treatment. Likewise, starting from week 6, DFMSC-treated mice showed significantly increased screen hang time than non-treated mice. CFA-immunized mice with or without DFMSC treatment did not show any signs of muscle weakness, as expected (Fig. 4).

### DFMSC administration ameliorates immunopathological findings of MuSK-related EAMG

**Fig. 5:**
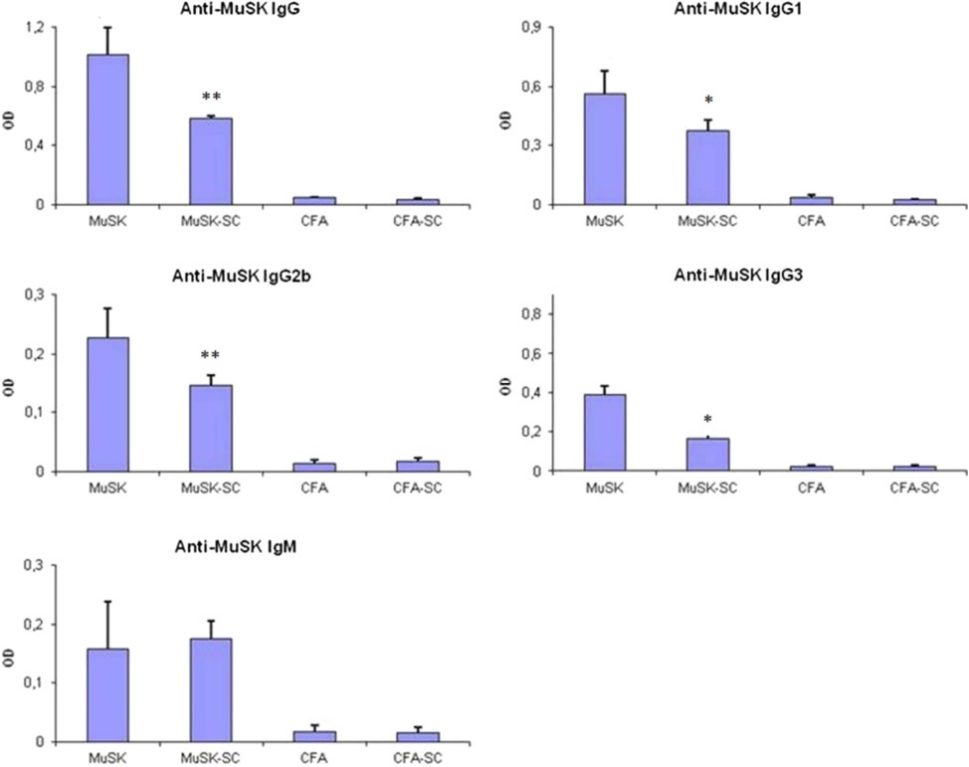
Serum anti-MuSK antibody levels in MuSK- and CFA-immunized mice with or without dental follicle mesenchymal stem cell (DFMSC) treatment. MuSK-immunized mice with DFMSC treatment (MuSK-SC) showed significantly lower anti-MuSK IgG, IgG1, IgG2b, and IgG3 levels than non-DFMSC-treated mice (MuSK). *Asterisks* only denote significant differences between MuSK and MuSK-SC groups as calculated by post hoc analysis. *p < 0.05; **p < 0.01 by ANOVA and Tukey’s post hoc test. *Vertical bars* indicate standard errors

**Fig. 6:**
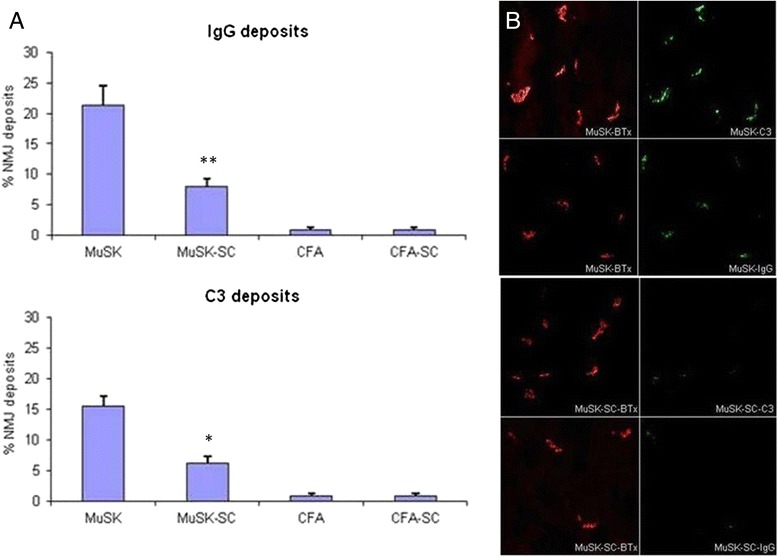
Assessment of frozen muscle samples of MuSK- and CFA-immunized mice with or without dental follicle mesenchymal stem cell (DFMSC) treatment by immunofluorescence studies showed reduced neuromuscular junction (NMJ) IgG and C3 deposit proportions in MuSK-immunized mice with DFMSC treatment (**a**). Representative pictures of muscle sections stained for C3 and IgG (*green fluorescence*) and bungarotoxin (BTx) for colocalization of the NMJs (*red fluorescence*) (magnification for all, ×100). The immunofluorescence data represent one of 5 sections for each mouse (**b**). *Asterisks* only denote significant differences between MuSK and MuSK-SC groups as calculated by post hoc analysis. *p < 0.05; **p < 0.01 by ANOVA and Tukey’s post hoc test. *Vertical bars* indicate standard errors

Tukey’s post hoc analysis indicated that MuSK-SC mice had significantly lower serum anti-MuSK IgG, IgG1, IgG2b, and IgG3 levels than MuSK-immunized mice with no DFMSC treatment, whereas anti-MuSK IgM levels of both MuSK groups were comparable (Fig. 5). Likewise, MuSK-SC mice were found out to display significantly lower percentages of NMJ IgG and C3 deposits than MuSK-immunized mice with no DFMSC treatment by Tukey’s post hoc analysis (Fig. 6a, b). Muscle sections of mice from CFA and CFA-SC groups did not show considerable NMJ deposits (not shown).

**Fig. 7:**
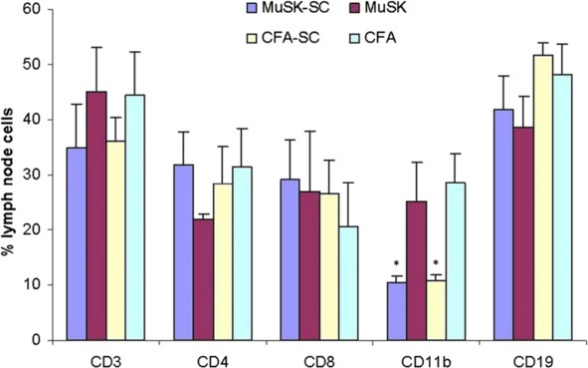
Lymph node cell proportions of MuSK- and CFA-immunized mice with or without dental follicle mesenchymal stem cell (DFMSC) treatment. MuSK-immunized and CFA-immunized mice treated with DFMSC showed significantly reduced CD11b+ cell percentages than non-SC-treated mice. *p < 0.05 by ANOVA and Tukey’s post hoc test. *Vertical bars* indicate standard errors

When lymph node cell subpopulations were analyzed by flow cytometry, CD11b+ cells were discovered to be significantly suppressed in both MuSK-SC and CFA-SC mice as compared to MuSK- or CFA-immunized mice with no DFMSC treatment. MuSK-SC and CFA-SC groups also showed trends towards reduced CD3+ cell proportions, although these differences did not attain statistical significance. There were no significant differences between percentages of CD4+, CD8+, and CD19+ cells among groups (Fig. 7).

### DFMSC administration suppresses proliferation and cytokine production responses of lymph node cells

**Fig. 8:**
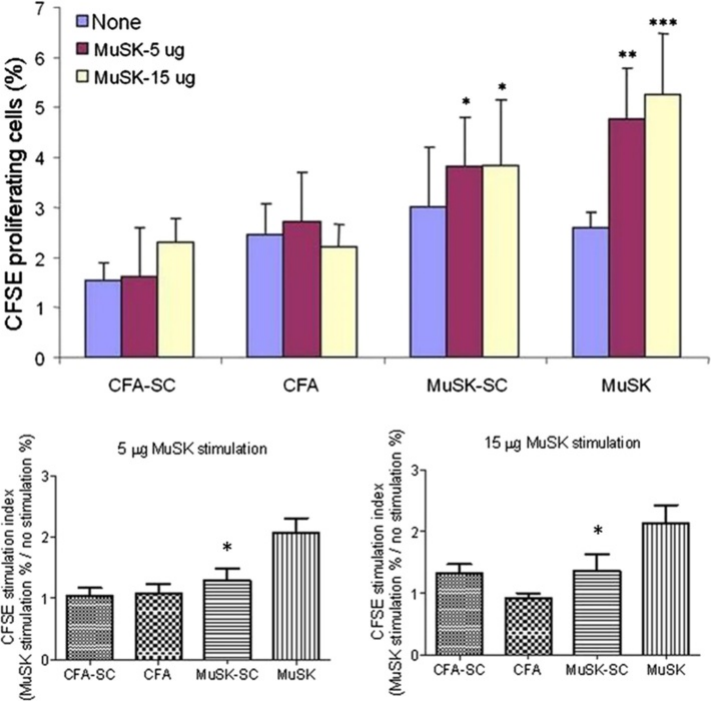
The percentage of proliferating cells in response to MuSK stimulation as measured by carboxyfluorescein succinimidyl ester (CFSE) labeling (*upper panel*). Lymph node cells of MuSK-immunized but not CFA-immunized mice showed significant proliferation by MuSK stimulation. Proliferation was less pronounced in MuSK-immunized mice treated with dental follicle mesenchymal stem cell (DFMSC) than those with no DFMSC treatment. Stimulation indices were analyzed by dividing lymph node cell percentages obtained by MuSK stimulation (*red* or *yellow bars*) by percentages obtained by no stimulation (*blue bars*). Only MuSK-immunized mice with no MSC treatment showed significantly increased indices, whereas MuSK-SC mice had comparable stimulation indices to CFA-immunized mice (*lower panels*). *p < 0.05; **p < 0.01; ***p < 0.001 by ANOVA and Tukey’s post hoc test. *Vertical bars* indicate standard errors

Lymph node cell proliferation in response to 5 and 15 μg of MuSK stimulation was measured by CFSE assay. Both MuSK and MuSK-SC groups showed proliferation responses, whereas no notable response was observed in CFA and CFA-SC groups, as expected. Lymph node cells of MuSK-immunized mice with no DFMSC treatment showed more distinct proliferation responses than those of MuSK-SC mice (Fig. 8, upper panel). Stimulation indexes were calculated by dividing proliferation values of MuSK-stimulated cells by proliferation values of non-stimulated cells. Stimulation indices of MuSK-SC mice were statistically comparable to those of CFA-immunized mice, whereas MuSK-immunized mice with no DFMSC treatment showed significantly higher stimulation indices than other immunization groups (*p* = 0.01 and *p* = 0.03 for 5 and 15 μg MuSK stimulations, respectively, by ANOVA). Tukey’s post hoc analysis showed significant differences for comparisons between the non-DFMSC-treated MuSK group and other groups (*p* < 0.05 for all comparisons) (Fig. 8, lower panels).

**Fig. 9:**
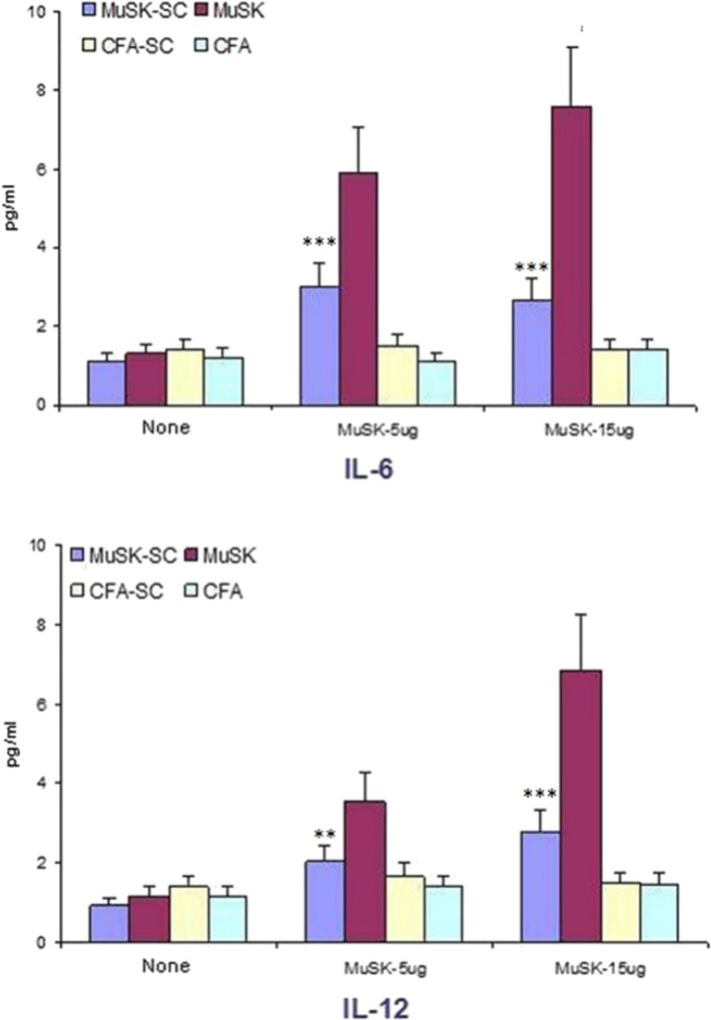
Cultured and MuSK- or non-stimulated lymph node cells of MuSK-immunized mice with no dental follicle mesenchymal stem cell (DFMSC) treatment showed significantly more IL-6 and IL-12 production than those of DFMSC-treated MuSK-immunized and CFA-immunized mice. **p < 0.01; ***p < 0.001 by ANOVA and Tukey’s post hoc test. *Vertical bars* indicate standard errors

Cytokine production responses to MuSK stimulation were also evaluated by measuring cytokine levels in supernatants of cultured lymph node cells. MuSK-immunized mice that were not treated with DFMSCs showed significantly higher IL-6 and IL-12 levels than other groups. Although MuSK-SC mice had also relatively higher IL-6 and IL-12 production levels than CFA-immunized mouse groups, these differences did not attain statistical significance (Fig. 9). There were no significant differences among MuSK-immunized mice with or without DFMSC treatment by means of supernatant levels of IL-4, IL-10, IL-13, IFN-γ, IL-21, IL-17, TGF-β, and TNF-α (not shown). Both MuSK-SC and non-DFMSC-treated MuSK-immunized mice showed significantly higher IL-4 and IL-10 levels than non-DFMSC-treated CFA and CFA-SC mice, whereas levels of other cytokines were comparable among all groups (not shown).

## Discussion

MSC administration has previously been shown to reduce clinical severity, serum antibody levels, and lymphocyte proliferation in the EAMG model induced by AChR immunization [21]. In this study, we have shown for the first time that DFMSC administration also ameliorates clinical and basic immunopathological findings of EAMG induced by MuSK immunization. In consistency with the previous report, DFMSC-treated mice did not only have lower clinical scores than untreated mice, but they also exhibited reduced anti-MuSK IgG levels, NMJ deposits, and lymph node cell proliferation capacity in response to MuSK stimulation. In line with EAMG studies, a single patient with AChR antibody-positive MG and motor neuronopathy has benefited from autologous bone marrow-derived MSC treatment [25], corroborating the notion that MSC may be effectively used as a potential future treatment method for MG patients.

In addition to previous EAMG studies, our studies also suggest that MSCs exert their beneficial effects presumably through suppression of CD11b+ cells. This marker is predominantly expressed by cells from the myeloid lineage and innate immune system displaying functions such as phagocytosis, antigen presentation, and neutrophil aggregation [26–29]. In the lymph node, CD11b is expressed by dendritic cells [27, 29, 30], which are known to play a crucial role in EAMG pathogenesis through presentation of NMJ antigens to lymphocytes and consequent activation of self-reactive T and B cells [31]. Therefore, as expected, treatment methods based on suppression of dendritic cell functions have effectively inhibited EAMG development in experimental models [31, 32]. Fcγ receptor knockout mice exhibiting impaired dendritic cell phagocytosis functions are known to be resistant to EAMG induction [33]. Moreover, DFMSCs have previously been shown in different animal models to preferentially inhibit cytokine secretion by dendritic cells and alter dendritic cell functions [34, 35]. It is thus tempting to speculate that DFMSC treatment ameliorates myasthenic muscle weakness through suppression of CD11b+ dendritic cells. However, CD11b is also expressed by monocytes, granulocytes, macrophages, and natural killer cells [26–29], all of which might potentially participate in EAMG pathogenesis. Therefore, exact significance of CD11b+ cell suppression and distinct cell populations involved in DFMSC-mediated EAMG amelioration need to be further studied through screening of an extensive panel of innate immunity markers by flow cytometry methods.

Notably, in our study, production of several cytokines primarily secreted by T and B cells were unaffected by DFMSC treatment. By contrast, the only two cytokines (IL-6 and IL-12) that were suppressed by DFMSC treatment are predominantly produced by myeloid cell lineage [36, 37]. Both IL-6 and IL-12 knockout mice have been shown to display significant resistance to EAMG induced by AChR immunization. Moreover, in resemblance to DFMSC-treated mice, both knockout mouse strains showed reduced antibody production, NMJ deposits, and lymphocyte proliferation capacity following EAMG induction [38, 39]. Although the significance of these two cytokines in MuSK-related MG is not well known, treatment methods based on IL-6 and IL-12 inhibition might presumably prove beneficial for MuSK antibody-positive MG patients, as well. Notably, despite exhibiting increased IL-4 and IL-10 levels as compared to CFA-immunized mice (as reported previously [40]), DFMSC-treated mice showed lower EAMG severity than non-DFMSC-treated mice, implying that non-Th2-type innate immunity also participates in MuSK-associated EAMG. Therefore, combined inhibition of Th2 immunity and the innate immune system might be required for effective treatment of MuSK antibody-positive MG patients.

## Conclusions

Although we have screened a broad panel of lymph node cells and cytokines, we have seen significant suppression only in very selective immune functions following DFMSC administration. Thus, overall, our findings emphasize that in contrast with immunosuppressants currently in use for MG management, DFMSC treatment has very specific effects on the immune system and nevertheless achieves a significant amelioration in clinical symptoms. This aspect of DFMSC treatment is particularly important in the management of MuSK antibody-positive MG patients, who often need to receive high dosages of cytotoxic medications. Our findings have also emphasized once again the importance of antigen-presenting cells and innate immune system cells in autoimmunity and suppression of these cells in treatment of autoimmune disorders. It is thus warranted to further study the impact of the interplay between different stem cell treatment models and innate immune system on MG pathogenesis.

## Additional file

Additional file 1: Table S1. Clinical incidences, average clinical grades, and inverted screen hang times of MuSK-immunized mice treated with different mesenchymal stem cell (MSC) types and cell numbers during optimization studies. The values were obtained at termination (28 days after the third MuSK immunization) and each experiment was done with 10 mice per mouse group. Note that lowest experimental autoimmune myasthenia gravis (EAMG) clinical scores were obtained with administration of dental follicle MSCs and with 1 × 10^6^ cells per injection (two injections in total). Immunization and MSC injection were done as described in the “Methods” section. (DOC 29 kb)

## Abbreviations

AChR: acetylcholine receptor
B6: C57BL/6
BTx: bungarotoxin
CFA: complete Freund’s adjuvant
CFSE: carboxyfluorescein succinimidyl ester
DFMSCs: dental follicle mesenchymal stem cells
DPSCs: dental pulp stem cells
EAMG: experimental autoimmune myasthenia gravis
MG: myasthenia gravis
MSC: mesenchymal stem cell
MuSK: muscle-specific tyrosine kinase
NMJ: neuromuscular junction
SHEDs: human exfoliated deciduous teeth
