# Supplementary material for: Dental follicle mesenchymal stem cell administration ameliorates muscle weakness in MuSK-immunized mice
Source: J Neuroinflammation. 2015 Dec 9;12:231. doi: 10.1186/s12974-015-0451-0 (PMC4673854; doi:10.1186/s12974-015-0451-0)
Supplement: Additional file 1: Table S1. — Clinical incidences, average clinical grades, and inverted screen hang times of MuSK-immunized mice treated with different mesenchymal stem cell (MSC) types and cell numbers during optimization studies. The values were obtained at termination (28 days after the third MuSK immunization) and each experiment was done with 10 mice per mouse group. Note that lowest experimental autoimmune myasthenia gravis (EAMG) clinical scores were obtained with administration of dental follicle MSCs and with 1 × 10^6^ cells per injection (two injections in total). Immunization and MSC injection were done as described in the “Methods” section. (DOC 29 kb) [file 12974_2015_451_MOESM1_ESM.doc]

**Supplementary Table 1.** Clinical incidences, average clinical grades and inverted screen hang times of MuSK-immunized mice treated with different mesenchymal stem cell (MSC) types and cell numbers during optimization studies. The values were obtained at termination (28 days after third MuSK immunization) and each experiment was done with 10 mice per mouse group. Note that lowest experimental autoimmune myasthenia gravis (EAMG) clinical scores were obtained with administration of dental follicle MSCs and with 1x10^6^ cells per injection (two injections in total). Immunization and MSC injection were done as described in Methods.

|  | EAMG incidence (%) | Average clinical grade (± standard error) | Average inverted screen hang time (± standard error) |
| --- | --- | --- | --- |
| Mouse compact bone MSC (2.5x10^5^ cells/each injection) | 60% | 2.2 ± 0.6 | 112 ± 15 seconds |
| Mouse compact bone MSC (1x10^6^ cells/each injection) | 40% | 1.7 ± 0.4 | 148 ± 25 seconds |
| Dental follicle MSC (2.5x10^5^ cells/each injection) | 50% | 1.9 ± 0.6 | 135 ± 17 seconds |
| Dental follicle MSC (1x10^6^ cells/each injection) | 40% | 1.6 ± 0.7 | 152 ± 32 seconds |
